# Impact of Functional Constipation on Health-Related Quality of Life in Preschool Children and Their Families in Xi’an, China

**DOI:** 10.1371/journal.pone.0077273

**Published:** 2013-10-10

**Authors:** Changjun Wang, Lei Shang, Yuhai Zhang, Jiao Tian, Baoxi Wang, Xianjun Yang, Lijun Sun, Chunyan Du, Xun Jiang, Yongyong Xu

**Affiliations:** 1 Department of Health Statistics, School of Public Health, Fourth Military Medical University, Xi’an, Shaanxi, China; 2 Department of Pediatrics, Tangdu Hospital, Fourth Military Medical University, Xi’an, Shaanxi, China; National Taiwan University Hospital, Taiwan

## Abstract

**Aim:**

Functional constipation (FC) is one of the common diseases among children. The aim of this study was to investigate the health-related quality of life (HRQOL) in preschool children diagnosed with FC and the impact of the condition on affected families.

**Methods:**

In this cross-sectional, case-control study, 152 children aged 3–6 years with FC, 176 healthy children aged 3–6 years without FC, and their primary caregivers were selected. Chinese versions of the PedsQL^TM^ 4.0 Generic Core Scale and the Family Impact Module (FIM) were used to assess childhood HRQOL and the impact of FC on family members, respectively. HRQOL scores were compared between children with FC and healthy children. In addition, a multiple step-wise regression with demographic variables of children and their caregivers, family economic status, duration and symptoms of FC, as independent variables, was used to determine factors that influenced HRQOL in children and had impacted caregivers.

**Results:**

Scores of physical, emotional, social and school functions, and summary scales were significantly lower in children with FC than in healthy children (*p* < 0.05). Physical, emotional, social, cognitive, and communication scores for caregivers, as well as daily activities and relationships for families of children with FC, were significantly lower than those of caregivers and families with healthy children (*p* < 0.05). Children’s ages, duration of FC, symptoms of FC, the child-caregiver relationship, family economic status, and caregiver education level emerged as the main factors influencing HRQOL in children, caregivers, and family members.

**Conclusions:**

FC had a significant impact on HRQOL of affected children and their caregivers, as well as their family functions. Social characteristics of children and caregivers, duration and symptoms of FC and family economic status significantly affected HRQOL of children and caregivers, as well as family functions of children with FC.

## Background

Constipation is a common childhood complaint. Its exact prevalence is difficult to ascertain because only a minority of patients suffering from constipation seek medical attention. In the hospital setting, constipation accounts for 3% of visits to pediatric clinics [1] and up to 25% of visits to pediatric gastroenterologists [2]. The hallmark symptom of constipation is infrequent defecation (less than three times per week), which is often painful. Unrecognized or inadequately treated constipation can lead to significant abdominal pain, appetite suppression, fecal incontinence, perineal infection/cellulitis, fissures, fistulae or tags with lowered self-esteem, social isolation and family disruption. Non-specific abdominal pain has been reported in 33% and painful defecation in up to 68% of children with constipation [1]. About 50% of children with chronic constipation are relieved of symptoms after a year and 65–70% after two years, with much higher relief rates in motivated families that adhere to treatment regimens [3]. About 34–37% remain constipated 3–12 years after treatment [3,4] and about 1/3 of constipated children continue to have constipation into adulthood despite treatment [5]. Families often report to care providers that the child’s constipation has taken over their lives.

Constipation rarely leads to life threatening complications, but can cause emotional and physical distress and concern for children and their families, ultimately impairing health related quality of life (HRQOL) [6-8]. The impact of constipation on both HRQOL of children and their caregivers is relevant to primary care providers, gastroenterologists and health care policy makers. About 90-95% of children’s constipation are functional constipation (FC) [9,10]; the prevalence of FC ranges from 0.7% to 29.6% (median 8.9; interquartile range 5.3–17.4) in children of both Western and non-Western countries [3,9-11]. But to our knowledge, the impact of FC on the HRQOL of children and the families has not been studied in an affected population of preschool children in China. The purpose of this study, therefore, was to evaluate the impact of preschool-period FC on children and their families, so as to provide scientific evidence for improving HRQOL of affected children and their families.

## Materials and Methods

### Diagnosis criteria for FC

FC has been defined by the ROME III classification [12,13]: (1) two or fewer defecations in the toilet per week; (2) at least one episode of fecal incontinence per week; (3) history of retentive posturing or excessive volitional stool retention; (4) history of painful or hard bowel movements; (5) presence of a large fecal mass in the rectum; and (6) history of large diameter stools which may obstruct the toilet. For infants and children less than four years of age, criteria must include at least two of the above for a month. Accompanying symptoms may include irritability, decreased appetite, and/or early satiety. For a child at least four years of age, criteria must include two or more of the above criteria with insufficient criteria for diagnosis of irritable bowel syndrome (IBS), and criteria should be met at least once per week for two months prior to diagnosis. 

Diagnosis of IBS requires two criteria [12,13] First, the patient experiences abdominal discomfort (uncomfortable sensation not described as pain) or pain associated with two or more of the following at least 25% of the time: (1) improvement following defecation; (2) onset associated with a change in frequency of stool; or (3) onset associated with a change in form (appearance) of stool. Second, the patient exhibits no evidence of an inflammatory, anatomic, metabolic, or neoplastic process that explains the symptoms. These criteria should be fulfilled at least once per week for two months prior to an IBS diagnosis.

### Data source

Between January and July 2011, 152 children (3–6 years old) with FC and their primary caregivers (parents or grandparents) were selected as the patient group from the pediatric outpatient departments of Xijing Hospital and Tangdu Hospital in Xi’an, China. The healthy control group included 176 healthy children (3−6 years old) and their primary caregivers (parents or grandparents) selected from the children’s health care centers at the same two hospitals. Healthy children were all being seen for routine annual wellness check-ups. All subjects had outpatient medical and health care records at one of the above-mentioned hospitals, and caregivers confirmed that the selected hospital was the primary medical or health care institution for the child. Primary caregiver was defined as the main caregiver who took care of the child’s daily living (diet, sleeping, activity, etc.) at home, after school, and during the weekend. Caregivers were asked whether he/she was the primary caregiver for the child; if he/she answered yes, that caregiver was asked to complete the survey.

Xijing Hospital and Tangdu Hospital are two of the largest AAA hospitals in Xi’an–one in the urban center, and the other in the suburbs. The AAA distinction means that they are among the best hospitals in China with the capacity to provide high-level medical services, and implement high-level medical education and research. Both hospitals treat a comprehensive spectrum of diseases and cover the whole population of Xi’an. The pediatrics departments provide service to more than 600 patients per day, and each has more than 200 beds for inpatients. The sub-specialties in the pediatric departments include respiratory diseases, digestive diseases, kidney diseases, and cardiovascular diseases.

For this study, patients had the following characteristics: 1) age 3−6 years old, 2) medical records at one of the designated study hospitals, 3) a diagnosis of FC, 4) nursery school or kindergarten attendance, and 5) during the preceding year, only study designated hospitals were used for health care/consultation. Child subjects were excluded from the study if they had other medical conditions such as organic causes of constipation (including Hirschsprung’s disease, muscle disorders, prior rectoanal surgery, spina bifida, mental retardation, or hypothyroidiosm), chronic diseases (e.g. asthma, heart diseases, renal disease, cancer, or epilepsy), neurodevelopmental disorders (e.g. autism spectrum disorder, dyspraxia, or conduct disorder), or growth abnormalities (e.g. dwarfism, failure to thrive, etc.) that influence HRQOL. Subjects were also excluded if their parents/caregivers were illiterate or unwilling to participate, were in poor health or had experienced severe negative events, such as a family member’s death, major accident, etc., in the preceding six months.

Members of the healthy control group met the following criteria: 1) age 3−6 years old, 2) health care records located at one of the designated study hospitals, 3) diagnosed by a pediatrician as developing normally, being healthy and free from FC for the past six months, 4) nursery school or kindergarten attendance, and 5) during the preceding year, only study designated hospitals were used for health care/consultation. The exclusion criteria applied to the control group were similar to those given to the patient group. Children who received disability or nutritional counseling were also excluded.

### Ethics statement

The Research Ethics Committee of the Fourth Military Medical University approved this study. All caregivers provided written informed consent. 

### HRQOL assessment

A Chinese version of the Pediatric Quality of Life Inventory™, version 4.0 (PedsQL^TM^4.0) was used in this study. The PedsQL^TM^4.0 consisted of three parts:

**1. PedsQL™ 4.0 Generic Core Scale**, developed by Varni et al. [14-16], has been cross-culturally adapted into Chinese by Hao et al. [17] and demonstrated good internal consistency, as well as discriminant and construct validity [17,18]. The Generic Core Scale is a brief, 23-item standardized questionnaire assessing a pediatric patient’s HRQOL, such that HRQOL is operationalized as the perceived impact of disease and treatment on a variety of functional domains (physical, emotional, social, and school function) [14,15]. Parents’ proxy-report and children’s self-reports determined the degree to which each item had been a problem for each child during the past month, using a 5-level scale: 0 = never a problem, 1 = almost never a problem, 2 = sometimes a problem, 3 = often a problem, and 4 = always a problem. Items were then linearly transformed to a 0−100 scale (0 = 100, 1 = 75, 2 = 50, 3 = 25, 4 = 0), so that higher scores indicated better HRQOL. The dimension scores were computed as the sum of the items divided by the number of items answered within a particular dimension, and the summary score was calculated as the sum of all 23 items divided by the number of items answered.
**2. The PedsQL^TM^ 4.0 Family Impact Module (FIM)** developed by Varni et al. [13,19] and cross-culturally adapted into Chinese by Chen et al. [20], demonstrated good internal consistency as well as discriminant and construct validity. The FIM is a parent-reported instrument that measures the impact of pediatric chronic health conditions on patient HRQOL and their family function. This 36-item instrument consists of eight dimensions: physical functions (six items), emotional functions (five items), social functions (four items), cognitive functions (five items), communication (three items), worry (five items), daily activities (three items), and family relationships (five items) [19]. The first four dimensions measure parent self-reported HRQOL, while the latter two dimensions measure parent-reported family functions. Likert-type scale responses were provided for each item: 0 = never a problem, 1 = almost never a problem, 2 = sometimes a problem, 3 = often a problem, and 4 = almost always a problem. Items were then linearly transformed to a 0−100 scale (0 = 100, 1 = 75, 2 = 50, 3 = 25, 4 = 0), meaning that higher scores indicated better HRQOL. The dimension scores were computed as the sum of the items divided by the number of items answered within a particular dimension. Three types of summary scores were calculated from FIM: 1) The summary score as the sum of all 36 items in the FIM divided by the number of items answered; 2) the parent HRQOL score as the sum of the 20 items from physical, emotional, social, and cognitive functions divided by the number of items answered; 3) the family function score as the sum of the eight items from daily activities and family relationship dimensions divided by the number of items answered.
**3. PedsQL™ Family Information Form**, developed by Varni [14,15], has been cross-culturally adapted into Chinese population and contains a child’s basic information (date of birth, gender, disease duration) and a caregiver’s basic information (marital status, occupation, family income level, and method of payment for the child’s medical care). In addition, a caregiver’s educational level, child-caregiver relationship, and characteristics of constipation (including duration of constipation, frequency of defecation, painful defecation, non-specific abdominal pain) were added to this form.

### Data collection and quality control

Five pediatric nurses, with at least five years of pediatric clinical nursing experience, performed this study. All investigators were trained prior to the survey so that they understood the survey purpose and its requirements. Caregivers completed the HRQOL questionnaires for the children and themselves including the PedsQL™ 4.0 Generic Core Scales, the PedsQL^TM^ 4.0 Family Impact Module and the PedsQL™ Family Information Form.

An investigator explained the purpose and details of all scales to the caregivers so that they could successfully complete the assessments. In addition, one of the main investigators carefully rechecked all the scales, and telephone interviews were conducted for any lost or incomplete information. 

### Statistical analysis

EpiData 3.1 software was used for data entry. To ensure data accuracy, double entry mode was selected and logistic errors were corrected.

Quantitative data were expressed as mean ± standard deviation, and qualitative data were expressed as percentages. A Chi-square (χ^2^) test was used to compare the distribution of children’s gender, caregiver education level, child-caregiver relationship, family economic status, and non-specific abdominal pain. Independent sample *t*-tests were used to determine differences in age, frequency of defecation, Generic Core Scales, and FIM scores between the two groups (FC vs. healthy). Pearson correlation coefficients (*r* values) were calculated to determine the relationship between scores of Generic Core Scale and FIM. Bonferroni corrections were applied to control multiple testing. Multiple step-wise regression analysis was used to select factors that influenced the HRQOL and family function in children and caregivers. Specifically, the influences of age (years), gender, caregiver education level, child-caregiver relationship, family economic status, duration of constipation (months), frequency of defecation (times/week), painful defecation, non-specific abdominal pain and HRQOL scores of caregiver or child on PedsQL™ 4.0 Generic Core Scales or FIM Scale dimensions were determined. Statistical significance was set at *p* < 0.05. All analyses were performed using the Statistical Package for Social Sciences software program, version 16.0 (SPSS, Inc., Chicago, IL, USA).

## Results

Demographic characteristics of the children and their caregivers are listed in Table 1. There were no significant differences in gender and mean age between children with FC and healthy children (all *p* > 0.05). Similarly, there were no significant differences in the proportion of child-caregiver relationships, caregiver education levels or family economical status between the two groups (all *p* > 0.05). The mean frequencies of defecation and non-specific abdominal pain were significantly different between the two groups (all *p* < 0.05).

**Table 1:** Baseline characteristics of children with FC and healthy children.

	children with FC (n = 152)		healthy children (n = 176)
Children’s gender (boys, %)	80 (52.6)		89 (50.6)
Children’s age ($\bar{x}\pm s$,years)	4.2 ± 0.9		4.3 ± 0.8
Child-caregiver relationship,n (%)			
Parents	108 (71.1)		124 (70.5)
Grandparents	44 (28.9)		52 (29.5)
Caregiver’s age ($\bar{x}\pm s$,years)	36.4 ± 4.2		37.1 ± 3.9
Caregiver’s education level (n (%))			
≤ 9 years	34 (22.4)		42 (23.9)
9–12 years	53 (34.8)		57 (32.4)
≥ 13 years	65 (42.8)		77 (43.7)
Family economic status (n (%))			
≤ 1500 yuan/individual·month	37 (24.3)		45 (25.6)
1500 ~ 3000 yuan/individual·month	60 (39.5)		68 (38.6)
≥ 3000 yuan/individual·month	55 (36.2)		63 (35.8)
Defecation frequency (times/week)	1.4 ± 0.5		5.5 ± 0.9^a^
Non-specific abdominal pain (n (%))	114 (75.0)		17 (11.2)^a^
Duration of constipation (months)	5.7 ± 2.3		_
Painful defecation occurrence (n (%))	82 (53.9)		_

Notes: (1) The difference in age and defecation frequency were analyzed using an independent sample *t* test; all others were analyzed using the χ^2^ test; (2) a:*p* < 0.05, Children with FC vs. healthy children.

The internal reliability estimates (Cronbach’s α coefficients) for summary scores and subscales of the Generic Core Scale and FIM Scale are presented in Table 2. For the Generic Core Scale, internal consistency estimates of the summary score had a value of 0.91 and subscale coefficients ranged from 0.73 to 0.89. For the FIM Scale, the internal consistency estimate of the summary score was 0.89 and subscale coefficients ranged from 0.83 to 0.90. Moderate to high item-summary correlations were observed across the scales.

**Table 2:** Internal consistency (Cronbach’s α Coefficient) and item-summary correlations of PedsQL^TM^ 4.0 Generic Core Scale and FIM Scale.

	Cronbach’s α Coefficient	Item-summary Correlations
**Generic Core Scale**		
Physical	0.89	0.53-0.71
Emotional	0.84	0.56-0.73
Social	0.79	0.51-0.69
School	0.73	0.47-0.61
Summary scale	0.91	0.55-0.72
**FIM Scale**		
Physical	0.90	0.62-0.85
Emotional	0.87	0.53-0.76
Social	0.85	0.59-0.82
Cognitive	0.84	0.52-0.73
Communication	0.80	0.56-0.70
Worry	0.85	0.59-0.72
Family daily activities	0.83	0.53-0.72
Family relationships	0.87	0.56-0.75
Summary score	0.89	0.50-0.68

Table 3 lists caregiver proxy-report scores for the Generic Core and FIM Scales. The scores on all dimensions of the Generic Core Scale among children with FC were significantly lower than those of healthy children (all *p* < 0.05). Children with FC had significantly lower scores on the FIM Scale than healthy children on each dimension except for “worry” (all *p* < 0.05).

**Table 3:** Mean scores ($\bar{x}\pm s$) of caregiver proxy-report on the Generic Core Scale and FIM Scale.

	Healthy children (n = 176)	Children with FC (n = 152)
**Generic Core Scales score**		
Physical	93.8 ± 8.8	77.4 ± 7.6^a^
Emotional	91.2 ± 9.3	64.3 ± 8.3^a^
Social	92.4 ± 8.0	72.3 ± 9.0^a^
School	89.4 ± 9.6	73.7 ± 10.2^a^
Summary score	92.1 ± 7.6	74.1 ± 6.9^a^
**FIM score**		
HRQOL	89.9 ± 7.8	64.7 ± 7.3^a^
Physical	91.4 ± 8.1	65.4 ± 9.4^a^
Emotional	89.6 ± 8.2	61.3 ± 8.6^a^
Social	90.9 ± 6.9	63.3 ± 7.7^a^
Cognitive	91.2 ± 6.3	64.6 ± 7.2^a^
Communication	92.3 ± 6.8	67.5 ± 9.1^a^
Worry	60.7 ± 8.1	88.9 ± 7.5^a^
Family Functioning	87.5 ± 9.2	62.6 ± 8.5^a^
Family daily activities	86.1 ± 7.6	59.8 ± 6.9^a^
Family relationships	89.4 ± 6.4	63.5 ± 6.3^a^
Summary score	88.2 ± 8.7	64.9 ± 9.4^a^

Notes: (1) Independent sample *t* test was used; (2) a*p* < 0.05 < FC children vs healthy children.

Table 4 lists the Pearson’s correlation coefficients between scores on the Generic Core Scale and FIM Scale. Higher scores on each dimension and summary score of the Generic Core Scale were associated with higher scores on the FIM Scale except for “worry” (all *p* < 0.05).

**Table 4:** The relationship between scores of the Generic Core Scale and FIM Scale.

FIM score	Score of the Generic Core Scale				
	Physical	Emotional	Social	School	Summary
HRQOL	0.56	0.52	0.48	0.57	0.61
Physical	0.54	0.48	0.46	0.49	0.56
Emotional	0.49	0.56	0.46	0.45	0.60
Social	0.43	0.55	0.50	0.54	0.57
Cognitive	0.39	0.48	0.43	0.44	0.45
Communication	0.44	0.38	0.45	0.43	0.45
Worry	-0.52	-0.53	-0.47	-0.39	-0.51
Family Functioning	0.48	0.54	0.42	0.45	0.47
Daily Activities	0.34	0.43	0.37	0.39	0.41
Relationships	0.53	0.44	0.38	0.46	0.48
Summary score	0.61	0.56	0.49	0.51	0.56

Notes: The data listed in table 4 are Pearson correlation coefficients between scores of FIM Scale and Generic Core Scale; all were statistically significant (*p* < 0.05).

Multiple step-wise analyses showed that child age, child-caregiver relationship, duration of constipation, frequency of defecation, painful defecation, non-specific abdominal pain, caregiver education level, caregiver HRQOL, and family economic status significantly influenced a child’s HRQOL summary score (all *p* < 0.05). Each dimension score on the Generic Core Scale was differentially affected by these variables (Table 5). 

**Table 5:** Factors influencing HRQOL in children with FC.

Factors	Score of the Generic Core Scale				
	Physical	Emotional	Social	School	Summary
Age (year)	0.14	0.32	—		0.21
Child - caregiver relationship	—	—	—	—	0.19
Caregiver education level 9–12 years	0.68	0.38	0.35	0.48	0.53
≥ 13 years	1.11	0.73	0.89	0.57	1.24
Caregiver’s HRQOL	0.48	0.35	0.32	—	0.36
Family economic status	—	0.26	0.15	—	0.16
Duration of constipation(months)	-0.23	-0.19	-0.39	-0.35	-0.39
Frequency of defecation(times/week)	0.36	0.41	0.26	0.29	0.42
Painful defecation occurrence	-1.26	-1.15	-0.88	-0.92	-1.28
Non-specific abdominal pain	-0.47	-0.30	-0.33	-0.27	-0.43
Determination coefficient *R* ^2^	0.76	0.49	0.57	0.58	0.73

Notes: (1) Multiple step-wise regression analysis was used to select factors that influenced the HRQOL in children with FC, using scores of physical, emotional, social, school, and the summary score of the Generic Core Scale as dependent variables. Age (years), gender, caregiver education level, child-caregiver relationship, family economic status, duration of constipation (months), frequency of defecation (times/week), painful defecation, non-specific abdominal pain and HRQOL scores were used as independent variables (2). The data listed in Table 5 was standardized partial regression coefficient; the ” — ” symbol indicates that this factor cannot enter the regression model. All standardized partial regression coefficients listed in the table were statistically significant (*p* < 0.05) (3). Caregiver’s HRQOL was the total score of the physical, emotional, social and cognitive scores of FIM.

Multiple step-wise regression analysis showed that child-caregiver relationship, duration of constipation, frequency of defecation, painful defecation, non-specific abdominal pain, caregiver education level, child HRQOL, and family economic status significantly influenced the summary score on the FIM Scale (all *p* < 0.05). Each dimension score of the FIM was differentially affected by these variables (Table 6).

**Table 6:** Factors influencing caregivers HRQOL and family function of children with FC.

Factors	Score of FIM scale								
	Physical	Emotional	Social	Cognitive	Communication	Worry	Daily Activities	Relationships	Summary
Age (year)	—	—	-0.31	—	-0.23	-0.39	-0.25	—	-0.36
Caregiver education level									
9–12 years	0.29	0.30	0.33	0.29	0.35	0.32	0.21	0.30	0.44
≥ 13 years	0.72	0.56	0.24	0.47	0.32	0.48	0.49	0.28	0.42
Child’s HRQOL	0.22	0.23	0.33	0.21	0.34	-0.21	0.43	0.54	0.69
Family economic status	—	0.26	0.15	—	—	—	0.37	—	0.26
Duration of constipation(months)	-0.19	-0.26	-0.24	-0.27	-0.23	0.28	-0.24	-0.28	-0.28
Frequency of defecation(times/week)	0.24	0.35	0.33	0.41	0.72	-0.31	0.49	—	1.02
Painful defecation occurrence	-1.09	-1.16	-1.24	-0.97	-1.23	1.09	-0.86	-0.98	-1.41
Non-specific abdominal pain	-0.79	-1.16	-0.64	-0.47	-0.53	0.99	-0.68	-0.39	-1.19
Determination coefficient *R* ^2^	0.66	0.36	0.47	0.28	0.51	0.76	0.46	0.44	0.64

Notes: (1) Multiple step-wise regression analysis was used to select factors that influenced HRQOL of caregivers and their family function, with scores of each subscale and summary scale as dependent variables, and with age (years), gender, caregiver education level, child-caregiver relationship, family economic status, duration of constipation (months), frequency of defecation (times/week), painful defecation, non-specific abdominal pain and HRQOL score of child as independent variables (2). The data listed in Table 6 were standardized partial regression coefficient and the “— ” symbol indicates that this factor cannot enter the regression model. All standardized partial regression coefficients listed in the table were statistically significant (*p*<0.05) (3). Child’s HRQOL was the summary score of PedsQL^TM^ 4.0 Generic Core Scale.

## Discussion

HRQOL is an important outcome measure in clinical trials, clinical improvement assessment strategies and population-based health assessment strategies [21]. Our results showed that HRQOL scores of physical, emotional, social, school functioning and a summary scale among children with FC were significantly lower than those of healthy children. Scores for physical, emotional, social, cognitive function, communication, daily activity, relationship and the summary score of FIM among families of children with FC were significantly lower than those of healthy children, except for the score for worry. Demographic variables of children and their caregivers, family economic status, duration of FC and symptoms of constipation were not only the key influencing factors to HRQOL of children with FC, but also to their caregiver QOL and family function. Therefore, impact of FC on HRQOL of children, their caregivers and their family functioning is significant. 

A few studies have focused on HRQOL in children suffering from FC [9,11,22,23]. In the United States, Youssef et al. [9] measured HRQOL using PedsQL^TM^ 4.0 Generic Core Scale in children aged 5–18 years with constipation in a case control model. Children with constipation had lower mean self-reported HRQOL scores and physical scores than healthy controls and children with inflammatory bowel disease (IBD) and gastroesophageal reflux disease (GERD). Parents of constipated children reported lower perceived emotional and social scores for their children compared to parents of healthy children and those with other chronic gastrointestinal diseases. In Australia [11], Clarke et al. measured HRQOL in 51 children aged 8–18 years with slow transit constipation (STC) using the PedsQL^TM^ 4.0 Generic Core Scale and found that total HRQOL scores were substantially lower in constipated children than their healthy peers, for both parent- and self-reported scores; STC appeared to impinge on both physical and psychosocial HRQOL. Faleiros and Machado [22] used the CHQ-PF50 to evaluate 57 Brazilian children aged 5–12 years with FC and 29 children with functional fecal retention, and compared them to 314 age-matched controls. They found decreased HRQOL scores both in psychological and physical well-being among the FC and the functional fecal retention groups, compared to the non-constipated, healthy children. Kinservik [24] assessed the repercussions of chronic constipation for QOL of 25 children aged 2–5 years and observed that the children and their families were negatively affected in physical, emotional and social aspects. We used the same questionnaire as Youssef et al. [9] and Clarke [11]. Our results were in accordance with Clarke’s in that the total QOL score, and physical and psychosocial QOL scores were substantially lower in constipated children than in their healthy peers. In contrast, Youssef et al. [9], Faleiros and Machado [22], and Kinservik [24] observed that not all the QOL scores were different between constipated children and healthy children. Our study found all the scores of domains and summary scores significantly lower in FC children than in healthy children. These differences may be due to the age range of subjects being studied, the questionnaire being used and measurements in our study being caregiver proxy-reports.

HRQOL was influenced by many factors. The duration and symptoms of diseases and social-economic characteristics of patients were the main factors influencing HRQOL. A study [23] in the Netherlands, which used the Defecation Disorder List (DDL) to assess QOL in 114 constipated children with fecal soiling, showed that both emotional and social domains of QOL were negatively affected and varied inversely with frequency of soiling episodes. Youssef et al. [9] found lower parent-proxy HRQOL scores that are mostly attributable to the duration of symptoms and family experience with constipation. They reported no significant differences in QOL between children with and without soiling. We did not survey fecal soiling. We found an association of painful defecation and non-specific abdominal pain with HRQOL of children, the duration of constipation was negatively correlated with HRQOL, while the frequency of defecation was positively associated with HRQOL of children. These results were similar to the findings in the study by Youssef et al. [9]. A study by Gerson et al. [25] on HRQOL of chronic kidney disease illustrated higher scores in physical, school, and social function on the PedsQL^TM^ 4.0 Generic Core Scale with higher maternal education level. The younger the patient is, the worse the score will be, across the dimensions. In our study, the effects of caregiver education level and child age on HRQOL were similar to correlation of these parameters in the study by Gerson et al. 

In pediatric chronic diseases, the impact of disease and treatment, on family functioning is a major concern [26-29]. Kristen et al. [30] found a significant, multi-domain impact of chronic pain on parental QOL; positive association of all FIM scales with pain catastrophe, functional disability, and emotional/behavioral problems; and inverse relation to pediatric QOL. Eiser et al. [31] found mothers of children newly diagnosed with cancer having significantly lower QOL than population norms, and mother’s QOL and worry being correlated with their children’s QOL. Williams et al. [32] found QOL in primary caregivers of children with asthma was positively correlated with QOL of those children; children with asthma had fewer days of school missed and lower asthma severity scores. Poverty impacts the QOL in families of children with disabilities by affecting health (e.g., hunger, limited health care access), productivity (e.g., delayed cognitive development, limited leisure opportunities), physical environment (e.g., overcrowded and unclean homes, unsafe neighborhoods), emotional well-being (e.g., increased stress, low self-esteem), and family interaction (e.g., inconsistent parenting, marital conflict over money) [33]. We found caregiver QOL correlated with HRQOL in children with FC, duration and symptoms of FC, child age, caregiver education level and family economic state.

The minimum age of the child to self-report HRQOL instruments varies from 7–9 years [34]. As our subjects were younger than 7 years old, we did not measure self-reported HRQOL. Parents’ perceptions of QOL for children with constipation, functional abdominal pain and inflammatory bowel disease are typically lower than their children’s self-reported scores [10,35,36]. The reasons for parents’ perceptions of a significantly impaired QOL could be the severity of the disease, frustration with the evaluation process and treatment modalities, or the parents’ personal experience with functional symptoms [37]. It is likely that parents or caregivers are worried about a child’s health status, whereas the child reports only obvious current problems.

The effects of medical interventions are reflected not only as changes in somatic parameters, but also in emotional and social aspects of patients’ lives. HRQOL assesses unexpected functional disability, monitors disease progression, and thus contributes to improvement of the prognosis. It is acknowledged as an essential health outcome measure in clinical trials, and in health services research and evaluation [38], and a relevant end-point for evaluating the efficacy of prevention measures, treatments, and rehabilitations in children [39]. HRQOL evaluations are critically informative in developing clinical disease-management programs that provide comprehensive treatment and education for ill children and their families. Using HRQOL as a foundation, pediatricians can develop targeted support services for children and their families, such as medical education about constipation and psychological counseling. Therefore, the ultimate goal of measuring HRQOL both in the affected children and in caregivers was to improve patient care by bridging the gap between care providers and their patients. If doctors can learn what questions to ask, and if children and their parents provide the right information, then this exchange of information might be more valuable than any vital sign or individual symptom.

We believe that our findings have an important value regarding HRQOL of children with FC. However, this study had several limitations too. First, HRQOL was assessed using PedsQL^TM^ 4.0 Generic Core Scale, which is a general questionnaire and thus may lack precision and sensitivity to identify important effects of distinctive symptoms of FC, such as fecal incontinence. But the scale was accepted by parents under non-supported conditions and had appropriate Chinese cultural adaptations. The use of generic instruments to evaluate HRQOL is indicated when subsets of children with the same problem are compared, or when comparing children with chronic conditions to healthy children. We did not have a gold standard for assessing the HRQOL of children with FC, therefore a specific instrument for the assessment of children with constipation and encopresis, like DDL, was proposed by Voskuijl et al. [36]. DDL has not yet been adopted in China. Second, the HRQOL results were obtained from children at large AAA hospitals in Xi’an and may not be representative of HRQOL of patients treated at different hospitals across different regions. But, our data do provide significant insight into the effect of FC on HRQOL, and we believe that it is useful to clinicians and researchers.

## Conclusion

FC is a frustrating symptom for children and their caregivers. Social characteristics of children and caregivers, duration and symptoms of FC, and family economic status significantly affected HRQOL and family function of affected children and caregivers. The lower HRQOL in these children, and high level of parental concern, create the need for more prompt management. Early recognition of symptoms and adequate treatments are necessary for successful outcomes. Therefore, it is important for problems identified in the HRQOL domains to be recognized and addressed as early as possible. HRQOL assessment can enable this recognition and may provide targets for additional (non-medical) intervention.
